# Hematogenous dissemination of pulmonary mucormycosis manifested as multiple subcutaneous nodules: a case report and review of the literature

**DOI:** 10.1186/s12879-022-07187-8

**Published:** 2022-03-04

**Authors:** Apiradee Taweesuk, Piriyaporn Chongtrakool, Panitta Sitthinamsuwan, Pakpoom Phoompoung

**Affiliations:** 1 Division of Infectious Diseases and Tropical Medicine, Department of Medicine, Faculty of Medicine Siriraj Hospital, Mahidol University, 2 Wanglang Road, Bangkoknoi, Bangkok, 10700 Thailand; 2 Department of Microbiology, Faculty of Medicine Siriraj Hospital, Mahidol University, Bangkok, Thailand; 3 Department of Pathology, Faculty of Medicine Siriraj Hospital, Mahidol University, Bangkok, Thailand

**Keywords:** Disseminated mucormycosis, Multiple subcutaneous nodules, Invasive fungal infection

## Abstract

**Background:**

Disseminated mucormycosis presenting with multiple subcutaneous nodules is a rare condition with a poor prognosis, and delayed diagnosis and treatment is common.

**Case presentation:**

We report a case of 64-year-old Thai woman with colorectal cancer who initially presented with *Acinetobacter baumannii* pneumonia and respiratory failure. Following 10 days after her admission to the intensive care unit, she developed hospital-acquired pneumonia. Five days later, multiple subcutaneous nodules appeared on both arms and both legs. Bronchoalveolar lavage and skin biopsy cultures both grew *Mucor* spp. She was diagnosed with disseminated mucormycosis and was treated with liposomal amphotericin B at a dose of 5 mg/kg/day. Despite treatment, our patient succumbed to septic shock and multiorgan failure on the third day after definitive diagnosis.

**Conclusions:**

This case demonstrates that the subcutaneous nodules caused by hematogenously disseminated mucormycosis are unusual in a patient with a solid tumor. Clinicians should be aware of this atypical presentation of mucormycosis in patients with solid tumors.

## Background

Mucormycosis is a fungal infection that is caused by different fungi of the order Mucorales. The predominant species, including *Rhizopus* spp., *Mucor* spp. and *Lichtheimia* spp., are typically found in decaying organic material and in soil [1]. Infection occurs via inhalation of sporangiospores, direct inoculation transmission, or ingestion [2]. The conditions that most strongly predispose a person to mucormycosis include diabetes mellitus with or without ketoacidosis, hematologic malignancies, solid organ and hematopoietic stem cell transplantation, corticosteroid use, trauma, iron overload, and malnourished status [3–5]. The disease is associated with extensive angioinvasion, thrombosis, tissue infarction and necrosis, and subsequent hematogenous dissemination of the fungi [3, 6]. The patterns of mucormycosis manifestation/involvement include rhino-orbito-cerebral, pulmonary, cutaneous, gastrointestinal, and/or disseminated infection. Of those, rhino-orbito-cerebral and pulmonary mucormycosis are the most prevalent types of mucormycosis [7].

Disseminated mucormycosis, which is defined as infection involving at least two non-contiguous sites, is the most severe type of mucormycosis, and it is associated with profound immunosuppression [8]. Disease dissemination occurs in up to 40% of patients with hematologic malignancies [9], but it is less common in patients with solid tumors [4]. Patients with disseminated mucormycosis may have secondary cutaneous involvement, with contiguous spreading from rhino-orbito-cerebral mucormycosis being the most common presentation, especially in diabetic patients [10]. Secondary cutaneous lesions resulting from hematogenous dissemination from other organs have only rarely been reported [11].

Here, we report a case of disseminated mucormycosis in a critically ill female patient diagnosed with solid tumor who presented with multiple skin lesions resulting from disseminated pulmonary mucormycosis. This is an unusual clinical presentation in a patient with this clinical and immunosuppression profile.

## Case presentation

A 64-year-old Thai woman presented at the emergency department of our center with fever, cough, and shortness of breath for 1 day. She had a history of hypertension, well-controlled asthma, and adenocarcinoma of the sigmoid colon stage 3. She underwent sigmoidectomy 4 months earlier, and completed the fourth cycle of adjuvant chemotherapy (capecitabine and oxaliplatin) 2 weeks earlier without infectious complication. At admission, the patient was febrile (body temperature 38 °C), and she had tachycardia, tachypnea, and hypotension. Her oxygen saturation was 70% on room air. Chest auscultation showed decreased breath sounds in the right upper lung field. The remainder of the examination was unremarkable. Chest X-ray showed right upper lobe consolidation. Laboratory investigations revealed hemoglobin of 8.8 g/dl; white blood cell count of 22,610/mm^3^ with 40% neutrophils, 2% lymphocytes, and 32% band forms; and, platelets of 188,000/mm^3^. Blood chemistry showed blood urea nitrogen of 31.5 mg/dl and creatinine of 1.03 mg/dl. Her liver function tests were within normal ranges.

She was admitted to the intensive care unit, and was diagnosed with severe community-acquired pneumonia with respiratory failure and septic shock. She was intubated, and was treated with intravenous meropenem, vasopressor, and hydrocortisone. Both sputum and blood cultures yielded *Acinetobacter baumannii* susceptible to carbapenems. On the third day after admission, she developed acute kidney injury which necessitated prompt initiation of continuous renal replacement therapy. Clinical improvement was then observed after 10 days of intravenous antibiotic therapy. Hydrocortisone for septic shock treatment was given at 200 mg per day for 10 days followed by tapering dose to complete 15 days of corticosteroid therapy. Her blood sugars were between 130 and 170 mg/dl.

**Fig. 1:**
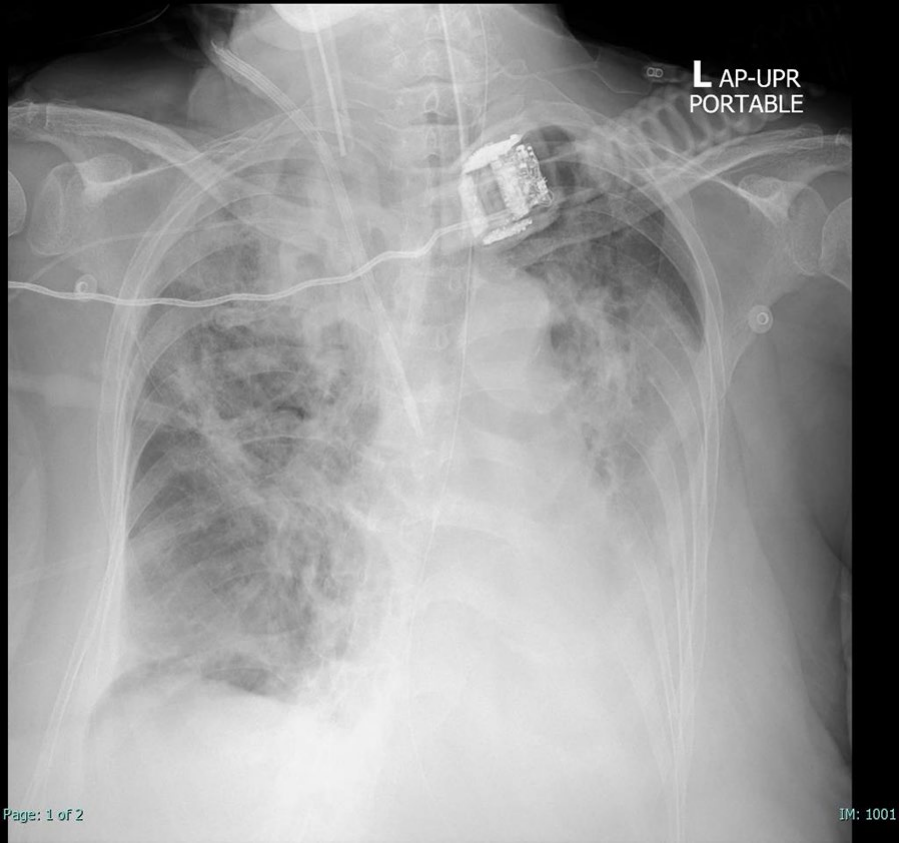
Plain radiograph of the chest on day 11 of hospital admission. Opacity at the right upper and left lower lung fields can be observed

However, her respiratory status worsened on the 11th day of admission despite hemodynamic stability. Chest X-ray showed new patchy infiltrations at the left lower lung field (Fig. 1). CT chest could not be performed due to her unstable respiratory conditions. Bronchoscopy revealed normal airway mucosa with purulent bloody secretions. Culture of the bronchoalveolar lavage (BAL) was positive for carbapenem-resistant *Klebsiella pneumoniae*. BAL culture for acid-fast bacilli was negative. BAL galactomannan was positive at an optical density of 4.01, but fungal culture was still pending. Serum galactomannan was negative. She was treated with intravenous fosfomycin, colistin, and voriconazole. The voriconazole was given as the empirical treatment for probable invasive aspergillosis due to the positivity of BAL galactomannan.

**Fig. 2:**
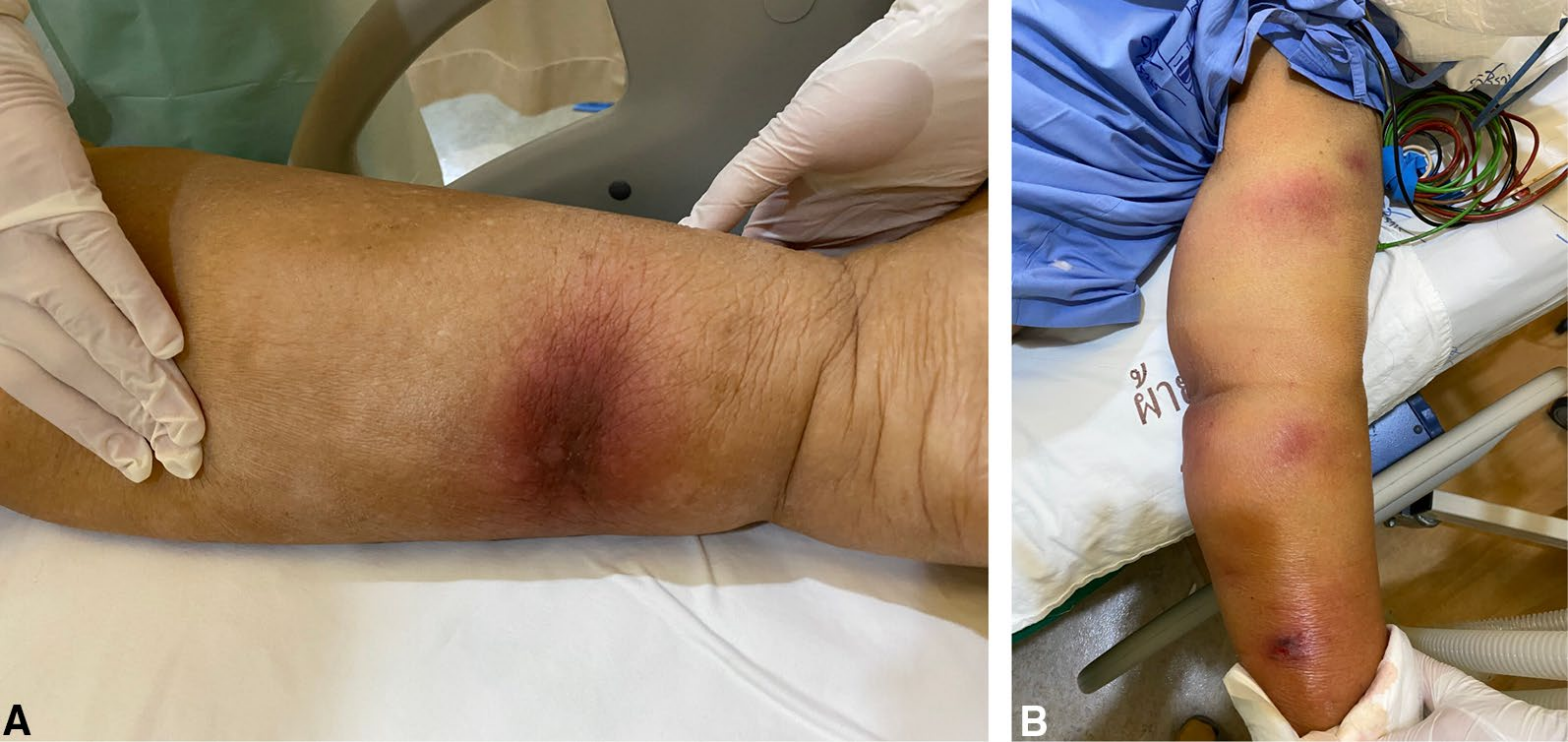
Multiple subcutaneous nodules on both arms and both legs (**A**–**B**)

**Fig. 3:**
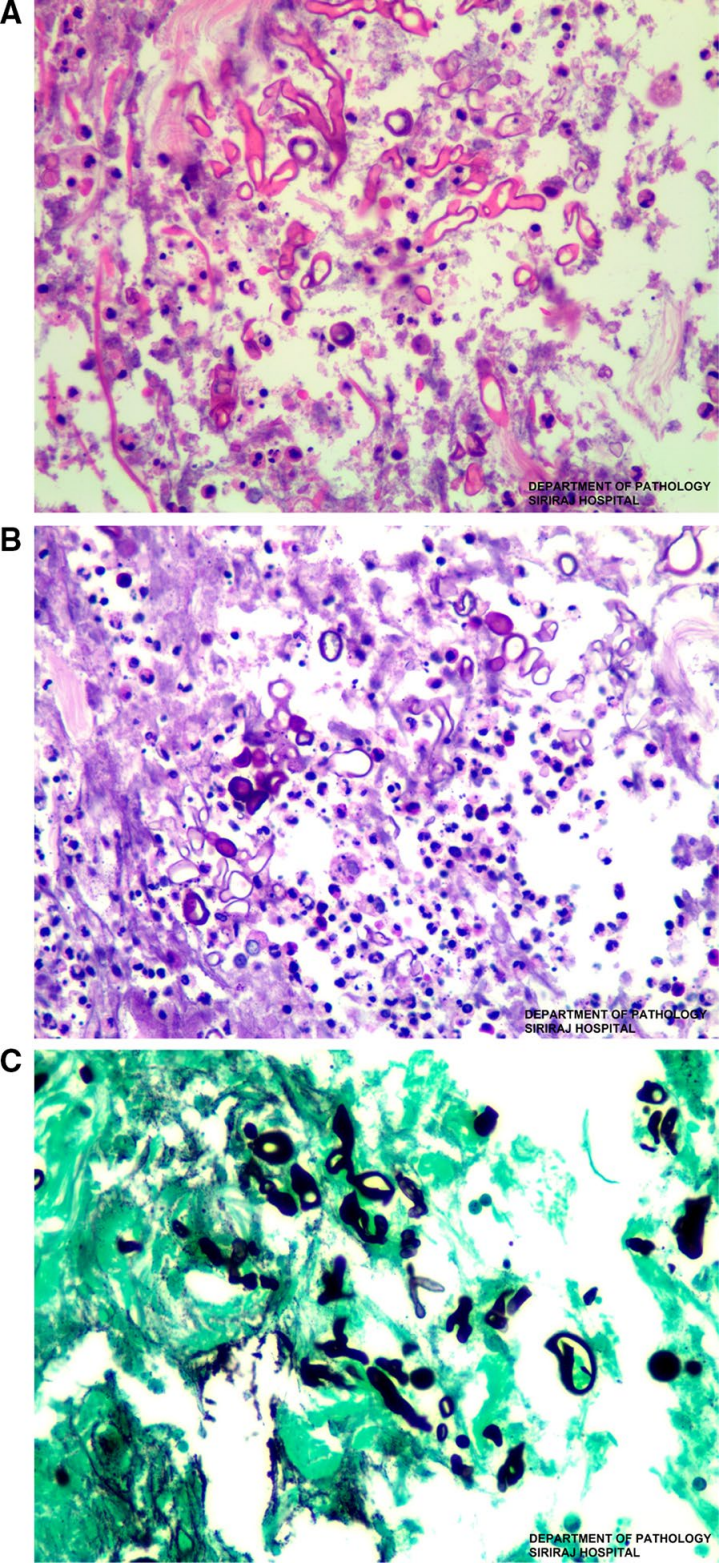
Hematoxylin and eosin (H&E × 400) stain (**A**), Periodic acid-Schiff (PAS × 400) stain (**B**) and Gomori methenamine silver (GMS × 400) stain (**C**). Skin biopsy of a subcutaneous nodule from the left arm showed broad rare septate broad hyphae with 90° branching

**Fig. 4:**
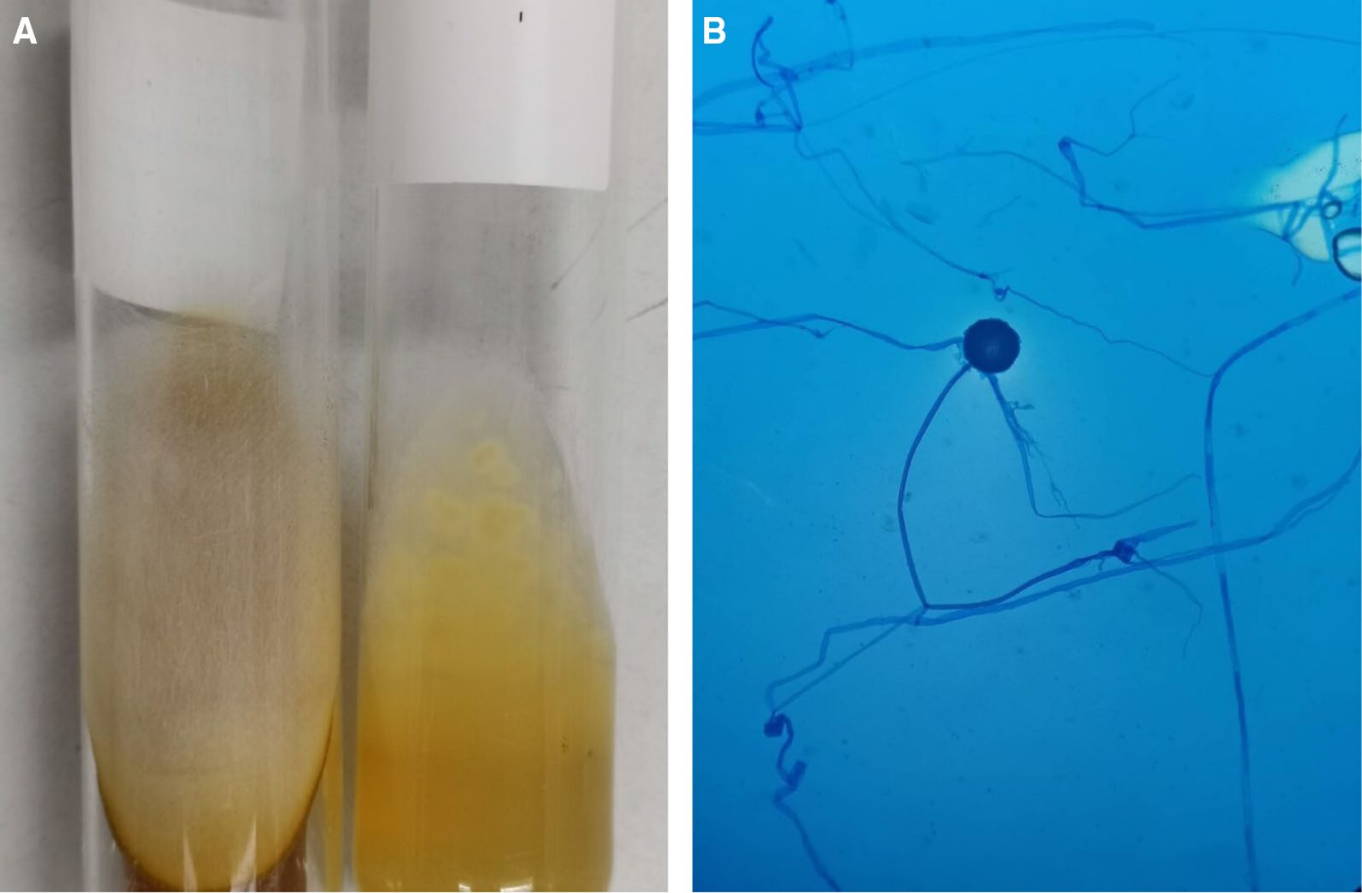
Macroscopic morphology showed a white to gray fluffy lollipop colony compatible with Mucorales (**A**). Microscopic morphology on lactophenol cotton blue staining showed spherical sporangium with no rhizoids (**B**)

On the 16th day of admission, after 1-day of hydrocortisone discontinuation, she developed multiple discrete ill-defined erythematous subcutaneous nodules on both arms and both legs (Fig. 2A, B). The lesions were 1–5 cm in diameter, and some were bullous lesions. To confirm diagnosis, a skin biopsy of a lesion on her left arm was performed. Histopathologic study of tissue sections revealed lobular panniculitis with suppurative granulomatous inflammation, and the presence of rare non-septate broad hyaline molds. Hematoxylin and Eosin (H&E) (Fig. 3A), Periodic acid-Schiff (PAS) (Fig. 3B) and Gomori methenamine silver (GMS) stains (Fig. 3C) showed positive for rare non-septate broad hyaline molds. Fungal culture of the skin biopsy revealed *Mucor* species. The fungal culture of the BAL specimen that was performed 5 days earlier also showed *Mucor* species (Fig. 4A, B). Fungal hemoculture was reported as negative result. She was then diagnosed with disseminated mucormycosis. Antifungal therapy was then changed to intravenous liposomal amphotericin B at a dose of 5 mg/kg/day. Despite treatment, our patient succumbed due to septic shock and multiorgan failure on the third day after definitive diagnosis.

## Discussion

The incidence of mucormycosis is increasing [12–14]**.** In the past, diabetes was acknowledged as a major risk factor; however, malignancy has emerged as another important risk factor [15, 16]. Some small cohort studies reported data on mucormycosis in severely ill patients [11, 17–20].

Cutaneous manifestation of mucormycosis could reflect either primary disease or secondary involvement from other organs. The common sites of involvement are the upper and lower extremities (46%) [4]. Primary cutaneous mucormycosis is often acquired via direct inoculation, and it occurs mainly in previously immunocompetent patients with massive soft tissue trauma, including burn injury, or in immunocompromised patients with minor skin trauma [21]. Skiada et al. [4, 21] and Roden et al. [22] reported that 40–50% of cases with cutaneous mucormycosis were immunocompetent. Typical lesions were indurated erythematous to purple plaques that could become necrotic and can develop into eschar [10]. Secondary cutaneous mucormycosis can result from rhino-cerebral disease with contiguous spread from the sino-orbital area, or from hematogenous dissemination from the other organs; however, the first mechanism has been more commonly reported. The most common cutaneous finding is a necrotic eschar, especially at the rhinofacial area [23]. In contrast, cases with hematogenous dissemination, such as our patient, have been only rarely reported in the literature. From a review of 929 reported cases of mucormycosis published before 2004, only 3% of cutaneous lesions were considered to be the result of dissemination to the skin [11]. Similarly, the largest literature review of cutaneous mucormycosis by Skiada et al. during 2004–2008 and 2009–2010 reported only one patient (1/119, 0.8%) with cutaneous mucormycosis resulting from hematogenous dissemination [4, 21]. In 2009, Dizbay, et al. reported the case of an 83-year-old woman with diabetes mellitus who was admitted to the intensive care unit [24]. She developed multiple erythematous nodules at her right hand, and the results of histopathology were compatible with mucormycosis. Hemoculture performed 6 days earlier and skin biopsy culture both grew *Mucor circinelloides*. She was treated with conventional amphotericin B; however, she died despite receiving appropriate antifungal treatment.

Comparing the immediately aforementioned case report with our patient, both patients were critically ill, and both had some degree of immunosuppression. Nevertheless, our patient received hydrocortisone at dosage of 2000 mg, which is equivalent to prednisolone at dosage of 500 mg. A previous study demonstrated that high corticosteroid use (> 600 mg) was associated with mucormycosis [25]. Recent multicenter study also showed corticosteroid treatment as important risk factor of mucormycosis in COVID-19 patients [26]. The outcomes were grave in both cases despite the administration of appropriate antifungal treatment.

Diagnosis of mucormycosis with cutaneous manifestation is quite difficult since most physicians are unfamiliar with the disease spectrum. Histopathology remains the standard diagnostic method. The biopsy specimen should be taken from the center of the lesion, and should include subcutaneous tissue because the fungi usually invade blood vessels in the deep cutaneous and subcutaneous layer [4]. Fungal culture is positive in only 50% of overall cases of mucormycosis. A previous study reported a higher rate of positive culture if the tissue was obtained from the skin and subcutaneous lesions (78%). Galactomannan has no significant benefit for diagnosing mucormycosis [27]. The positive BAL galactomannan in our patient was quite high. We hypothesized that it could be the result of mixed fungal infection or *Aspergillus* colonization.

Treatment of mucormycosis consists of early antifungal treatment, adequate surgery, and reversal of immunosuppression [27]. The recent guidelines describe liposomal amphotericin B as the current first-line therapy [28, 29]. Radical surgery is crucial in patients with locally extensive cutaneous involvement; however, role of surgical debridement in patients with multiple cutaneous lesions remains unclear.

The significance of the case described relates to an episode of disseminated mucor, including skin manifestations, in a patient with a history of solid tumor and recent prolonged steroid use. Thus, the presence of subcutaneous nodules with bleb or necrosis in a critically ill patient should prompt further investigation to exclude mucormycosis as the possible cause.

## Conclusions

Disseminated mucormycosis can occur in critically ill patients. Cutaneous involvement as a result of disseminated mucormycosis is a rare, but serious disease. Early diagnosis and appropriate antifungal therapy are needed to improve patient outcome.

## Data Availability

All data generated during this study are included in this published article.

## Abbreviations

BAL: Bronchoalveolar lavage
H&E: Hematoxylin and eosin
PAS: Periodic acid-Schiff
GMS: Gomori methenamine silver
